# Exploring Postoperative Cognitive Dysfunction and Delirium in Noncardiac Surgery Using MRI: A Systematic Review

**DOI:** 10.1155/2018/1281657

**Published:** 2018-03-18

**Authors:** Chenxi Huang, Johan Mårtensson, Ismail Gögenur, Mohammad Sohail Asghar

**Affiliations:** ^1^Department of Surgery, Center for Surgical Science, Zealand University Hospital, Copenhagen University Hospital, Køge, Denmark; ^2^Department of Clinical Sciences, Lund University, Lund, Sweden; ^3^Department of Neuroanaesthesia and Intensive Care, Rigshospitalet, Copenhagen University Hospital, Copenhagen, Denmark

## Abstract

Surgical patients are at high risk of developing postoperative cognitive dysfunction (POCD) and postoperative delirium (POD). POCD and POD are associated with increased morbidity and mortality and worsening functional outcomes leading to severe socioeconomic consequences for the patient and the society in general. Magnetic resonance imaging (MRI) offers a unique opportunity to study the anatomy and function of the brain. MRI thus plays an important role in elucidating the neuronal component of POCD and POD. Our aim has been to systematically gather MRI findings that are related to POCD and POD. Systematic searches were conducted in PubMed, EMBASE, and PsycINFO: MRI studies investigating patients with POCD as identified by perioperative cognitive testing or patients with delirium identified postoperatively by the Confusion Assessment Method. A total of ten eligible papers were included with a total of 269 surgical patients, 36 patient controls, and 55 healthy controls who all underwent MRI examination. These studies suggested that reduction of thalamic and hippocampal volumes and reduction of cerebral blood flow may be associated with POCD, while presurgery/preexisting and postoperative white matter pathology may be associated with POD. However, the evidence from these studies is rather weak. Future MRI studies are warranted to verify the current findings.

## 1. Introduction

Approximately a quarter billion major surgical procedures are performed every year worldwide [1]. Cognitive impairment arising after surgical intervention is referred to as postoperative cognitive dysfunction (POCD) and is a common adverse event to surgery. The manifestations of POCD may be subtle and diverse, depending on which cognitive domain that is affected. Most often, memory, intellectual performance, and/or executive functions are affected [2]. It is well established that cardiac surgery can cause transient or permanent POCD [3, 4]. However, POCD can occur after any kind of surgery [5, 6]. A larger multicenter study showed that 25.8% of noncardiac surgical patients suffered from POCD one week after the surgery, and 40% of these patients had persisting cognitive dysfunction three months after surgery [5]. POCD is associated with increased mortality and risk of leaving occupation prematurely [7]. POCD may subsequently lead to mild cognitive impairment and dementia even in patients who have recovered from the initial cognitive decline [8, 9].

In addition to POCD, elderly surgical patients are especially at high risk of developing postoperative delirium (POD) immediately after the surgery [10]. POD is characterized by an acutely altered mental state with features of inattention, disorganized thinking, and fluctuating level of consciousness, representing acute brain dysfunction. Some of these features are overlapping with POCD, as inattention and disorganized thinking affect normal memory and executive functions. The onset and course of POCD and POD are, however, distinctive. POD often sets in 24–72 hours after surgery with a reversible course of fluctuating severity, whereas POCD is often observed weeks after surgery and persists for longer time [11]. Although often overlooked by clinicians, POD is a serious condition associated with poor outcome including longer hospitalization, increased early mortality, and POCD [12, 13]. Because of the rising mean life expectancy in Europe and in the US, it is expected that more and more elderly patients will undergo surgery, and thus many more surgical patients will develop POCD and POD in the years to come.

Even though cognitive impairment after anesthesia and surgery was described as early as in 1955 [14], the pathophysiology of POCD and of POD is still poorly understood. It has been suggested that surgery-induced tissue damage activates peripheral immune system facilitating inflammatory responses that causes neuroinflammation and degeneration [15–17]. Although intraoperative anesthetics are mostly untraceable a few days after the surgery, some studies have suggested that anesthetic drugs may facilitate POD and POCD by suppression of neurogenesis [6, 18]. In cardiac surgery, silent cerebral infarctions are particularly evident. Cerebral infarctions may contribute to the development of POCD and POD [19]. In noncardiac surgery, silent cerebral infarctions are less common [20], it is therefore believed that the development of POCD and POD is related to surgical stress [21].

Advancements of neuroimaging, particularly, in magnetic resonance imaging (MRI), provide useful tools to noninvasively measure neuronal activity and capture detailed images of the human brain [22, 23]. MRI offers a unique opportunity to study minute and subtle neuroplastic changes in the brain in relation to changes in physiology and disease [24]. MRI may thus be used to elucidate neuropathological aspects of POCD and POD. Findings of MRI correlates of POCD and POD can potentially help clinicians to identify patients in risk, reducing the postoperative mortality.

Acute systemic inflammatory response triggered by surgery initiates immune activations that cause deterioration in cognition and disturbance in attention which result in higher morbidity and mortality. The aim of this systematic review has been to gather the existing literature of MRI findings associated with those cognitive deficits in order to improve diagnosis and treatment of POD and POCD. MRI findings can serve as biomarkers that may determine severity of the cognitive impairment, identify those patients that are responsive to specific treatment, and monitor the efficiency of the treatment.

## 2. Materials and Methods

In POCD, memory and executive function are most often affected [25], but to date, no consensus has been reached for which cognitive tests there should be used or what threshold for cognitive decline should be employed to define POCD [26]. POD is diagnostically defined according to the criteria stated in The Diagnostic and Statistical Manual of Mental Disorders or ICD-10. However, in perioperative research, rapid symptom rating score with a validated method such as the Confusion Assessment Method (CAM) is more readily applicable [27]. Accordingly, we have predefined that for studies to be eligible for inclusion they had to fulfill the following criteria: (i) With regard to POCD studies, pre- and/or postsurgery neuropsychological testing of attention, memory, or executive functions should have been performed within 30 days of surgery, (ii) with regard to delirium studies, delirium identified within 30 days of surgery by CAM or validated CAM based delirium screening tools (e.g. 3D CAM and CAM-ICU), and (iii) all studies should contain a population that have undergone surgery prior to the occurrence of POCD or POD. Furthermore, all included studies had to report at least one MRI finding (regardless whether it was obtained from functional or structural imaging) and had to contain original data of adults (≥ 18 years) published in English in a peer-reviewed journal (no protocol articles, conference abstracts, case reports, or duplicate publications). Exclusion criteria were neurosurgeries, cardiac surgeries, liver or kidney surgeries, and bariatric surgeries due to their association with cerebral surgical trauma, cerebral micro-infarctions, pre-existing encephalopathy, weight loss and diet-related neuroplasticity, respectively [4, 28, 29].

Systematic searches were conducted in three major databases at 27th September 2017 (PubMed, EMBASE, and PsycINFO). The following key words were used in combination for eligible studies: “MRI” OR “BOLD” OR “DTI” OR “Resting state” AND “Surgery” AND “Cognitive” OR “Cognitive dysfunction” OR “Cognitive decline” OR “Cognitive deficits” OR “Delirium.” No filters were applied.

One author (CH) extracted the following data from included studies whilst a second author (MSA) verified the extracted data. The authors then separately reviewed the search results at title and abstracts levels with regard to the inclusion and exclusion criteria. In case of disagreement between the authors, consensus was reached within the author group for a final decision. The studies selected for full-text review were then evaluated by both authors (CH and MSA) as described earlier. In case of disagreement between the authors, consensus was reached within the author group for a final decision. Information was extracted from each included study: characteristics of population (including age and gender), type of surgical intervention, outcomes of delirium or cognitive tests and MRI data, and their correlations to delirium or cognitive outcomes. This study was performed in accordance with the PRISMA guidelines for systematic reviews and has been registered in PROSPERO database (registration number: CRD42015023738) [30].

The methodological quality of the included studies was assessed by the authors (CH and MSA) using Newcastle Ottawa Scale for case control studies [31]. It is comprised of 8 items within three broad perspectives: the selection of study groups; the comparability of the groups; and the ascertainment of the exposure of interest. A star is given for each item if methodological criteria is met, apart from item comparability, in which two stars are given. Each study can be given a maximum of 9 stars representing the highest quality.

## 3. Results

**Figure 1:**
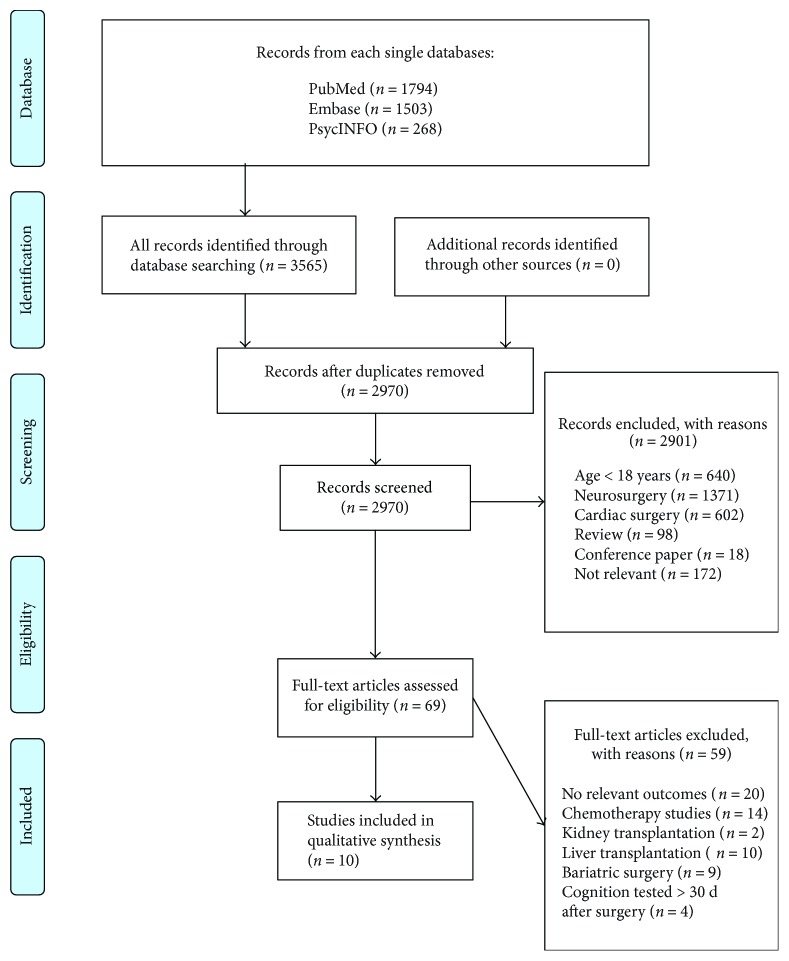
Flowchart of study selection.

Searches provided a total of 3565 papers. After eliminating all duplicate papers, 2970 papers were screened at title and abstract levels. Sixty-nine papers were selected for full-text evaluation, of which 59 papers were excluded (Figure 1). In total, ten eligible papers were included in this review [10, 32–40], of which nine were prospective studies (three case-control studies and one cross-sectional study reporting six different MRI endpoints), and one retrospective study (case-control). These studies included a total population of total 269 surgical patients, 36 patient controls and 55 healthy controls. Six of the papers reported different MRI endpoints of the same large population consisting of 146 patients who underwent mixed surgery (hip or knee replacement and colectomy) [10, 32–34, 36, 38]. The remaining studies comprise 23 patients who underwent partial lung resection [39], 31 patients who underwent total knee arthroplasty [37], 30 patients who underwent breast cancer surgery [40], and finally 39 patients who underwent open abdominal surgery [35]. Overview of included studies is presented in Tables 1 and 2, whereas Table 3 summarizes the principle findings. The methodological quality of the included studies is shown in Table 4. The methodological quality is overall good, receiving between four to eight stars in Newcastle Ottawa Scale. The majority of the included studies had a fair or good methodological quality due to good comparability (accounting for several confounding factors), clearly defined and well-represented cases, and same method of ascertainment for cases and controls. No study received a star for nonresponse rate due to either lack of report or unequal dropout rate.

### 3.1. Postoperative Cognitive Decline

Three blinded studies compared presurgery structural differences by MRI in patients with POCD to controls [35, 37, 40]. In a cross-sectional study by Chen et al., cognitive tests were performed the day before surgery and repeated four days after surgery in patients undergoing open abdominal surgery [35]. Of the 39 patients included, 36% (*N* = 13) was diagnosed with POCD. Using 1.5 Tesla MRI, the authors reported a 6% reduction in hippocampal volume (*p* < 0.01) in the POCD group compared to the non-POCD group. The authors also reported that hippocampal volume was negatively correlated to score of POCD (B = − 1.940, beta = 0.346, *p* = 0.044). Price et al. included 31 surgical patients undergoing total knee arthroplasty and 12 age- and education-matched patient controls with osteoarthritis. In this case-control study, tests of cognitive function were performed 2 weeks before surgery and again at 3 weeks, 3 months, and 1 year after surgery [37]. The rates of POCD in terms of memory decline were 17%, 25%, and 9%, at 3 weeks, 3 months, and 1 year, respectively. In terms of executive function decline, the rates were 21%, 22%, and 9% at 3 weeks, 3 months, and 1 year, respectively. 3T MRI scans were performed within 2 weeks after the surgery. The study showed declines in executive and memory functions at 3 weeks in the surgery group (*p* < 0.05); however, these declines were not associated with hippocampal volume (beta = −0.18, *p* > 0.05) in a regression model. However, volumes of leukoaraiosis and lacunae (>2 mm) were correlated to the memory declines at 3 weeks and at 1 year postsurgery (beta = −0.22, *p* < 0.027). In addition, the corrected total brain volume was also associated with executive function decline (but not with executive function directly) at all time periods (beta = −0.23, *p* = 0.03). In a prospective study by Sato et al. including 30 females undergoing surgery for breast cancer and 20 age-matched postmenopausal healthy controls, MRI measurements at 3-tesla were performed at baseline (one day before surgery in the surgery group) and repeated after five days [40]. In addition, a battery of cognitive function assessments was performed at baseline and repeated within 1 week for both groups. A lower score in the cognitive test, Digit Cancellation Task, was recorded in the patient group after surgery compared to controls (*p* = 0.001), reflecting postoperative cognitive decline. A concomitant significant reduction in the right thalamic volume from pre- to postsurgery was also recorded in the surgery group and was correlated to the cognitive decline (*p* < 0.05).

### 3.2. Postoperative Delirium

Seven recent studies have investigated neural correlates of POD in patients without dementia [10, 32–34, 36, 38, 39]. In a retrospective case-control study of brain metastasis-free patients undergoing pneumonectomy or lobectomy due to non-small-cell lung cancer, a set of delirium cases was identified retrospectively by a chart-based CAM review of medical records [39]. In total, 23 patients diagnosed with POD and additional 24 age- and gender-matched patient controls without delirium were included. 1.5T MRI scans (T1 and FLAIR) were performed prior to surgery. The patients with delirium had significantly greater degree of white matter hyperintensity compared to age- and gender-matched patient controls without delirium (F(3) = 6.144, *p* = 0.017). There was no association between cerebral atrophy and delirium (*p* = 0.118). In a prospective cross-sectional study using 3T MRI by Saczynski et al. of a mixed surgical population (*N* = 566, predominately hip/knee replacement and colectomy), the authors reported that 24% of all patients developed CAM-verified POD [10]. Anatomical MRI scans were performed on a subset of patients (*N* = 146) prior to surgery to exclude brain metastasis. Thirty-two (21%) of these 146 patients developed POD. Post hoc analysis did not reveal a correlation between intracranial volume and risk ratio of POD (RR = 1.41 (0.60–3.32)). Further MRI analysis of imaging data from subsets of the same study population has been reported in five separate publications [32–34, 36, 38]. In the first publication, the authors failed to find any significant correlation between relative risk of delirium incidence and total volume of cerebral white matter hyperintensity (RR = 0.966 (0.919–1.016), *p* = 0.18), brain parenchymal volume (global brain atrophy) (RR = 0.997, *p* = 0.367), or hippocampal volume (RR = 1.37, *p* = 0.545), both with and without adjustment for age, gender, and cognitive performance (*p* values stated here were for the adjusted models) [34]. Hippocampal volume or brain parenchymal volume were neither correlated to the severity of POD (*p* = 0.686 and *p* = 0.795, resp.); however, white matter hyperintensity was correlated to delirium severity (*p* = 0.045). In the second publication, the presurgery diffusion tensor imaging (DTI) findings (fractional anisotropy and axial, mean, and radial diffusivity) were reported [32]. DTI is sensitive to microstructural damage to white matter. DTI scans were performed in 136 of the original 146 patients who underwent MRI scans. POD occurred in 29 (21%) of these patients. The study found that the general cognitive performance at baseline was significantly lower in patients that developed POD (*p* = 0.003). Presurgery white matter abnormalities (decreased fractional anisotropy and increased axial, mean, and radial diffusivity) in several brain regions were found to be associated with delirium incidence and severity after controlling for age, gender, and vascular comorbidities (*p* < 0.001–0.05). After additional controlling for general cognitive performance, only the white matter abnormalities of the cerebellum, hippocampus, thalamus, and basal forebrain remained associated with delirium incidence and severity (*p* = 0.001–0.015). In the follow-up study, the DTI measurements were repeated one year after surgery in most of the patients from the original patient group (*n* = 113, of which POD had occurred in 25 patients (~22%)) [33]. At the one year follow-up, cognitive performance remained significantly lower in the delirium group (*p* = 0.02). The delirium severity was associated with decrease in fractional anisotropy (FA) and increase in mean diffusivity (MD) (*p* < 0.05), primarily in white matter of the frontal, parietal, and temporal lobes, with slight right-sided lateralization. In addition, the changes in cognitive performance over the year were associated with decrease in FA and increase in MD. This was predominantly found in the white matter of the posterior temporal, parietal, and occipital lobes (*p* < 0.05). In the fourth publication, the results from the arterial spin labeling (ASL) measurements were reported. Here, whole brain cerebral blood flow (CBF) measurements were performed before the surgery in the original MRI cohort (*n* = 146) [36]. The study did not find any significant association between global or voxel-wise CBF and delirium incidence (*p* > 0.05) or severity (*p* > 0.05). Stated as secondary endpoints, the global CBF and regional CBF in parietal lobe were both correlated to cognitive performance tests including trails B (*p* = 0.034) and digit symbol substitution (*p* = 0.049). In the fifth and final publication, the investigators calculated the cortical thickness in mm of nine predefined brain regions related to Alzheimer's disease in 145 patients (of the 146 patients who underwent presurgical MRI scans) [38]. The cortical thickness was not associated with risk of delirium (OR = 1.15 (0.84–1.57), *p* = 0.38), but is was inversely associated with delirium severity in a regression model (coefficient −1.2 (−2.2 to −0.27), *p* = 0.014). In addition, the cortical thickness was smaller in the predefined superior parietal region in patients who developed delirium (*p* = 0.018). The remaining eight brain regions did not show any significant effects (*p* = 0.16–0.98).

## 4. Discussion

We have reviewed all studies that we could find that investigated MRI findings of alterations of the brain in relation to POCD and POD in noncardiac surgical populations. The utilization of neuroimaging within this field is still limited, and the evidence is sparse; a total of ten observational studies were eligible for inclusion in the present review.

### 4.1. Postoperative Cognitive Decline

The studies indicated that reduced volume of the thalamus, reduced volume of the hippocampus, preexisting degenerative white matter pathology (leukoaraiosis or lacunae) or reduced CBF may be correlated with POCD. However, the evidence from these studies is rather weak.

POCD often includes a decline in memory and executive function. These brain functions are primarily governed by higher order cortices (e.g., prefrontal cortex and temporal lobes), with the thalamus acting as a relay for the information between these cortical areas [41]. In particular, the dorsomedial thalamic nucleus is critical for obtaining new information and for executive functions [42]. Lesions here lead to impaired attention and reduced speed of information processing [43]. In several neurological diseases, such as stroke, the thalamus is also found to be associated with cognitive impairment [44]. It is therefore plausible that the executive dysfunction seen in POCD may be caused by a reduction in functional connectivity to the thalamus or by a reduced thalamic volume. This is supported by Graff-Radford et al., who found that atrophy of thalamus was correlated with POCD after abdominal surgery [44]. Scherling et al. in a fMRI study of women undergoing mastectomy showed that altered frontal and thalamic activation patterns were associated with slower working memory-related reaction [42].

A reduction in hippocampal volume has not only been reported in an abdominal surgical population in relation to POCD. Similar findings have been reported after cardiac surgery [45]. Hippocampus plays an important role in memory and is critical for working memory function and for memory consolidation [46, 47]. In a large neuroimaging database, Kline et al. found that atrophy of the hippocampus was accelerated months after noncardiac surgery, which was also correlated with persistent POCD [15]. In addition, two large prospective MRI database studies found accelerated brain atrophy five to ten years after noncardiac surgery [48, 49].

The structural and functional changes may be a result of neuroinflammation. The normal aging brain, including the hippocampus and thalamus, is more susceptible to cognitive impairments following immune challenges such as infection and surgery, effects that are due to age-induced hyper-sensitization of the microglia cells in the brain [50]. In addition, surgery induces an upregulation of proinflammatory factors in the central nervous system and in peripheral tissues [51]. A recent meta-analysis even showed significant correlation between elevated peripheral inflammatory marker, primarily IL-6 and S-110Beta, and the occurrence of POCD [52]. One MRI study showed a disruption of the blood-brain barrier within 24 hours of surgery. This disruption may contribute to an increased inflow of proinflammatory cells into the brain [53].

Finally, studies indicate that nonspecific changes in cerebral white matter (e.g., hyperintensities or lacunar infarcts) and reduced cerebral blood flow are associated with increased risk of cognitive decline as well as Alzheimer's disease [36, 54, 55]. It seems that surgery induces alteration in specific brain regions that are related to memory and executive function and initiates accelerated long-term brain atrophy.

### 4.2. Postoperative Delirium

The studies concerning POD reported mostly presurgery MRI data and only one study provided longitudinal data. Most of the studies were arisen from the Successful Aging after Elective Surgery (SAGES) cohort study designed to examine novel risk factors and long-term outcomes associated with delirium. Different MRI modalities and analysis methods had been applied to the same cohort of 146 elective surgical patients. Using DTI analysis, it was possible to find an association between preexisting and postsurgery white matter microstructure abnormalities in several brain regions and POD incidence and severity [32]; however, other MRI measurements (cortical thickness, cerebral blood flow, global brain atrophy, total volume of white matter hyperintensity, and hippocampal volume) were not found to be associated with the incidence of POD in the same cohort [10, 34]. Interestingly, in another much smaller patient group, POD has been found to correlate with total volume of white matter hyperintensities [39]. The studies included in current review did carefully exclude confounding factors (e.g., patients with dementia). In particular, the SAGES studies did also control for other important confounding factors (cognitive status, age, gender, and vascular comorbidities) [10, 32–34, 36, 38]. Despite this effort, the SAGES studies suffer from lack of a priori endpoints. In addition, a major caveat is that four of the publications are based on presurgery data from the very same population. The results are thus of explorative nature, and further prospective studies are needed to verify the findings.

Two recent neuroimaging reviews investigated delirium in nonsurgical settings including patients with stroke, psychiatric patients, and intensive care unit (ICU) patients. Total volume of white matter hyperintensities, cortical atrophies, subcortical atrophies, and reduced cerebral blood flow, particularly in the parietal and frontal regions, were found across these patient groups in relation to delirium [56, 57]. Specifically, in psychiatric patients treated with electroconvulsive therapy, white matter hyperintensity was pronouncedly found in periventricular and deep regions. Furthermore, Morandi et al. recently reported that the fractional anisotropy of major white matter tracts (corpus callosum and internal capsule) was reduced in ICU patients with delirium at time of discharge correlated to longer delirium episodes at the ICU [58, 59]. In addition, Choi et al. reported that the resting state functional connectivity (rsfMRI) between the ascending reticular activating system (which regulates arousal and consciousness) and subcortical structures (the interlaminar thalamic nuclei, striatum, and caudate) was reduced in patients diagnosed with delirium during hospitalization due to various medical conditions compared to after recovery and compared to healthy controls [60]. A diminished connectivity may explain the decrease in orientation and alertness during acute delirium. Interestingly, this study also found changes in intrinsic connectivity networks (via rsfMRI) that had some similarities with patients in vegetative state or in healthy subjects during propofol-induced unconsciousness. This may thus be related to the attention deficit that persist beyond the amelioration of the acute delirium symptoms [60]. The current data suggest that high volume of preexisting white matter hyperintensities, which was also found in various nonsurgical patients and patients with delirium after cardiac surgery, may be associated with POD. There are, however, discrepancies in affected brain regions as well as the nature of the white matter lesions. Exceptionally, the thalamus is seemingly often affected across various conditions reflecting its important role in delirium.

### 4.3. Limitations

The majority of the included studies had a low risk of bias according to Newcastle Ottawa Scale; however, these studies still have limitations such as diversity of design and patient population between the studies and small sample sizes. In terms of MRI methodology, the majority of the MRI modalities employed in the included studies were confined to coarse structural comparison either as analysis of global volume changes or region-of-interest (ROI) analysis of a limited few regions (i.e., hippocampus and thalamus). Unfortunately, only five studies have applied more advanced methods (CBF, VBM, and DTI) to investigate changes in brain regions in relation to POCD and POD [32, 36, 40]. Furthermore, the unspecific imaging findings such as white matter hyperintensity and leukoaraiosis in patients with POCD are difficult to interpret [61], as these findings can be found in the aging brain of healthy individuals, although leukoaraiosis has a higher prevalence in patients with dementia (40%). Compared to cardiac surgery, the clinical significance of silent ischemic lesions after noncardiac surgery in POCD remains controversial [20]. However, a recent study reported an incidence as high as 10% of silent infarctions after noncardiac surgery [62], indicating that the changes in cerebral hemodynamics that leads to ischemic lesions may also play an important role in POCD. In addition, it has been speculated that neuroinflammation caused by postoperative changes in the immune response also contribute to the pathophysiology of POCD.

**Figure 2:**
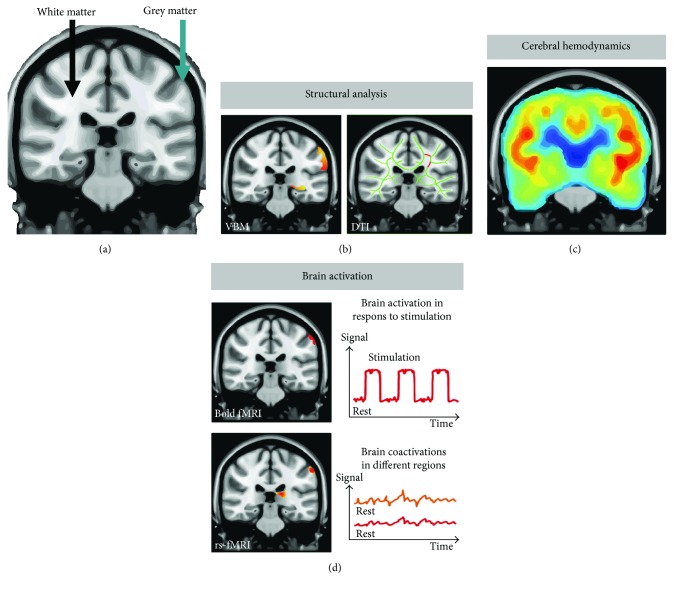
A brief overview of MRI modalities to elucidate cerebral pathophysiology in relation to POCD and POD. (a) Anatomical MRI scan showing white matter and grey matter of the brain. (b) (Left side) Voxel-based morphometry (VBM) analyses the anatomical images to determine possible changes in grey matter volume and morphometry. (b) (Right side) Diffusion tension imaging (DTI) can characterize white matter integrity and tracts. These modalities can potentially reveal even minor neuroplasticity. (c) Measurement of cerebral hemodynamics with intravenous (IV) contrast permits determination of, for example, cerebral blood flow (CBF), cerebral blood volume (CBV), and cerebral oxidative metabolic rate (CMRO_2_), while CBF also can be measured without IV contrast by arterial spin labeling (ASL). (d) Functional MRI can indirectly determine neuronal activation by measuring concomitant changes in blood flow. Blood-oxygenation-level dependent (BOLD) fMRI signal is useful in investigating brain activation to an explicit task. Resting state fMRI (rsfMRI) can reveal coactivation of distinct regions across the brain in patients that are not performing an explicit task.

There are several MRI modalities that may further elucidate changes in the brain in response to surgical stress (Figure 2). Voxel-based morphometry (VBM) and surface-based analysis (SBA) of anatomical MRI data permit detection of subtle changes in especially the grey matter [63, 64]. Diffusion tension imaging (DTI) utilizes diffusion of water molecules in white matter to enable white matter tracking and detect changes in neural pathways between brain centers (e.g., track-based spatial statistics (TBSS) or tractography) [65, 66]. Functional MRI (fMRI) offers insight in understanding brain activation using blood-oxygenation-level dependent (BOLD); fMRI is an indirect way of measuring neuronal activity by recording concomitant changes in blood flow, which can be used to investigate brain activation to cognitive task that reveals impairment of attention or cognitive dysfunction. Resting state fMRI provides a method of functional imaging that can be used to evaluate cerebral interregional interactions when the subject is idle (not performing a task). Therefore, future studies hold many promising opportunities to investigate cerebral changes in relation to POCD and POD.

## 5. Conclusion

In conclusion, this review indicates shortage of MRI neuroimaging studies investigating the neural bases of POCD and POD after noncardiac surgery. In light of their limitations, no firm conclusion can be drawn; however, the included studies showed indications of both preexiting/presurgery brain vulnerability and postsurgery white and grey matter alterations were related to postoperative delirium and cognitive decline. Both delirium and cognitive decline are commonly seen among noncardiac surgical patients, and they can potentially result in poor outcome and death. Elucidating the neural basis of POCD and POD after noncardiac surgery, both in terms of structural and functional aspects of neuroplasticity, may enable clinicians to provide preventive care and treatment strategies to these vulnerable patients. Future studies using advanced MRI methodologies and serial imaging are thus highly warranted.

## Figures and Tables

**Table 1:** Overview of included postoperative cognitive dysfunction (POCD) studies.

Study	Design	Surgery	Patients and controls (age)	Cognitive test (timing)	MRI system	MRI timing	MRI modalities	Statistics	Major findings
Sato et al. [40]	Prospective, case-control	Breast cancer surgery	30 patients (60 ± 7) chemonaïve postmenopausal 20 healthy controls (59 ± 5)	Digital span back Stroop color word test Wechsler adult memory scale Digit symbol coding Digit cancellation task (1/2 days before surgery, within 1 week of surgery)	3.0T Philips	1x presurgery 1x postsurgery	T1 weighted VBM	ANCOVA multiple regression	Attention domain subtest lower score in patients compared to control after 1 week. Significant group by time interaction in the right thalamus (*p* < 0.05) Correlation between change ratio of digit cancellation task and decrease of thalamus rGMV

Price et al. [37]	Prospective, case-control	Total knee arthroplasty (TKA)	31 patients (70.8 ± 7.0) Charlson comorbidity 1 + 1 12 arthritis patient controls, age and education matched (72.2 ± 5.3) Charlson comorbidity 1.7 + 1.8	Hopkins verbal learning, story memory, brief visual spatial memory, digital span back, spatial span subset, digit symbol substitution, controlled oral word association, Stroop color word test, category fluency, judgment of line orientation, and finger tapping test (baseline, 3 wk, 3 mo, 1 yr)	3.0T Siemens	1x presurgery	T1 weighted	Hierarchical regression models	Preoperative leukoaraiosis and lacunae volume improve the prediction model of baseline Stroop color word test of 3 wk postoperative executive function decline, but not with memory decline

Chen et al. [35]	Prospective, cross-sectionalstudy	Open abdominal surgery	39 patients (72 ± 8) ASA 2-3 35 controls (71 ± 5)	Wechsler adult memory scale and intelligence scale, trail making grooved pegboard (1 day before and 3 days after surgery)	1.5T Siemens	1x presurgery	T1 weighted	POCD (13) versus non-POCD (23)	6% reduction of hippocampus volume in POCD group compared to non-POCD, this reduction is correlated negatively with *Z* score

**Table 2:** Overview of included postoperative delirium (POD) studies.

Study	Design	Surgery	Patients and controls (age)	Delirium test (timing)	MRI system	MRI timing	MRI modalities	Statistics	Major findings
Racine et al. [38]	Prospective, cross-sectionalstudy	Mixed surgery	145 patients (76.7 ± 5.2), Charlson comorbidity-all range	CAM (during postoperative hospital stay once pr. day)	3T GE	1x presurgery	Cortical thickness	*T*-test GML	The cortical thickness did not predict occurrences of delirium in these patients; however, among those who had delirium, the thinner cortical thickness was associated with worse delirium severity.

Cavallari et al. [33]	Prospective, cross-sectionalstudy	Mixed surgery	113 patients (76.7 ± 5.2), Charlson comorbidity-all range	CAM (during postoperative hospital stay once pr. day)	3T GE	1x presurgery 1x postsurgery	DTI protocol	GML	The delirium severity was positively and negatively associated with fractional anisotropy (FA) and mean diffusivity (DM), respectively, and primarily in white matter of the frontal, parietal, and temporal lobes

Hshieh et al. [36]	Prospective, cross-sectionalstudy	Mixed surgery	146 patients (76.7 ± 5.2), Charlson comorbidity-all range	CAM (during postoperative hospital stay once pr. day)	3T GE	1x presurgery	ASL-derived CBF	Multiple regression models	No significant differences in whole brain CBF nor in the globally normalized voxel wise analysis. CBF in parietal lobe is correlated to cognitive performance

Cavallari et al. [32]	Prospective, cross-sectionalstudy	Mixed surgery	136 patients (76.7 ± 5.2), Charlson comorbidity-all range	CAM (during postoperative hospital stay once pr. day)	3T GE	1x presurgery	DTI protocol	*T*-test GML	DTI abnormality in cerebellum, hippocampus, basal forebrain, thalamus, ant commissure

Cavallari et al. [34]	Prospective, cross-sectionalstudy	Mixed surgery	146 patients (76.7 ± 5.2), Charlson comorbidity-all range	CAM (during postoperative hospital stay once pr. day)	3T GE	1x presurgery	T2 weighted and FLAIR	Multiple regression models	No significant differences in white matter hypersensitivity, global brain atrophy, and hippocampal volume

Saczynski et al. [10]	Prospective, cross-sectionalstudy	Mixed surgery	146 patients (76.7 ± 5.2), Charlson comorbidity-all range	CAM (during postoperative hospital stay once pr. day)	3T GE	1x presurgery	T2 weighted and FLAIR	Multiple regression models	24% of total patients had delirium, no correlation between intracranial volume (ICV) and POD

Root et al. [39]	Retrospective, case-controlstudy	Partial lung resection	23 lung cancer patients with delirium (73.4, 54.0–86.0) 24 patient controls age and sex matched without delirium (73.6, 54.0–85.0)	Retrospective medical record review in regard to CAM (within 4 day of the surgery)	1.5T GE	1x presurgery	T1 weighted and FLAIR	Delirium versus nondelirium (ANCOVA)	Higher white matter hyperintensity significantly associated with POD

**Table 3:** Overview of MRI findings in postoperative cognitive dysfunction (POCD) and delirium (POD), respectively.

Investigated MRI correlates	POCD	POD
Hippocampus volume	Reduced [35]	No difference [34]
White matter hyperintensity (bright areas on images seen in normal aging or neurological disease)	Increased [35]	Increased [39] No difference [34]
Lacune (cerebrospinal fluid filled cavities in white matter, seen in normal aging or neurological disease)	Increased [37]	N/A
Intracranial volume (ICV) (total volume within the cranial borders)	N/A	No difference [10]
Total parenchymal volume (total brain volume (grey and white matter))	N/A	No difference [34]
Cerebral blood flow (blood perfusion in the brain, hypoperfusion is for example related to the pathogenesis of Alzheimer's disease)	Reduced [36]	No difference [36]
Thalamus volume	Reduced [40]	N/A
White matter integrity (DTI abnormalities, mean and radial diffusivity, axial diffusivity, and fractional anisotropy)	Reduced [33]	Reduced [32, 33]

**Table 4:** Newcastle Ottawa Scale of included studies.

Study	The selection of the study groups	The comparability of the groups	The ascertainment of the exposure of interest
Racine et al. [38]	^☆☆☆☆^	^☆☆^	^☆☆^
Cavallari et al. [33]	^☆☆☆☆^	^☆☆^	^☆☆^
Root et al. [39]	^☆☆^	^☆^	^☆^
Saczynski et al. [10]	^☆☆☆☆^	^☆☆^	^☆☆^
Cavallari et al. [40]	^☆☆☆☆^	^☆☆^	^☆☆^
Cavallari et al. [32]	^☆☆☆☆^	^☆☆^	^☆☆^
Hshieh et al. [36]	^☆☆☆☆^	^☆☆^	^☆☆^
Chen et al. [35]	^☆☆☆^	^☆☆^	^☆^
Price et al. [37]	^☆☆☆^	^☆☆^	^☆☆^
Sato et al. [40]	^☆☆☆☆^	^☆☆^	^☆☆^
